# P02.82. Correction of the Omega-3 Index in women with metabolic syndrome by adding omega-3 supplements to a Mediterranean-style diet

**DOI:** 10.1186/1472-6882-12-S1-P138

**Published:** 2012-06-12

**Authors:** R Lerman, K Browning, L Kaskel, M McIntosh, W Najm, M Fernandez, E Baruffi, W Harris

**Affiliations:** 1 Metagenics Inc., Gig Harbor, USA; 2 Browning Family Medical Therapeutic Lifestyle Center, Riverside, USA; 3 Deerpath Primary Care, Libertyville, USA; 4 Dept. of Emergency Medicine, University of Florida, Jacksonville, USA; 5 Dept. of Medicine, University of California, Irvine, Irvine, USA; 6 Dept. of Nutritional Sciences, University of Connecticut, Storrs, USA; 7 Sanford School of Medicine, University of South Dakota, Sioux Falls, USA

## Purpose

To determine the effects of a Mediterranean-style, low-glycemic-load (MLG) diet with and without omega-3 fatty acid supplementation on the Omega-3 Index (O3I) in women with metabolic syndrome.

## Methods

One trial and one case series are reported here. The trial was a 12-week multicenter study (N=56) testing the effects of MLG diet on the O3I, and the case series was conducted in the offices of two physicians and included 21 women who were given, in addition to a MLG diet recommendation, one of two omega-3 supplements for approximately 12 weeks. One supplement (N=12) provided 1980 mg EPA+DHA per day (High DHA, Metagenics Inc.), and the other supplement (N=9) provided 2880 mg EPA+DHA daily (EPA-DHA 720, Metagenics Inc.). RBC fatty acid profiles were determined by gas chromatography and CVD risk factors by standard laboratory methods.

## Results

In the clinical trial with the MLG diet alone, the O3I rose by 14.9% [from 4.35% to 5.00% (p< 0.0001)], due largely to an increase in fish intake. RBC trans fatty acid, linoleic acid, and alpha-linolenic acid levels decreased. In the case series, in the 12 subjects who received High DHA, the O3I rose by 104.2% [from 3.6% to 7.3% (p<0.001)] whereas in the 9 subjects who consumed EPA-DHA 720, the O3I rose by 99.8% [from 4.2% to 8.3% (p<0.001)]. The increase in the O3I per g of EPA+DHA given per day was 1.88% with High DHA and 1.44% with EPA-DHA 720.

## Conclusion

The MLG diet led to a small improvement in the O3I but target levels were not achieved. Provision of 2-3 g/day of EPA+DHA for 12 weeks increased the O3I by 3.7 - 4.1 percentage points and was sufficient to raise the O3I to cardioprotective levels. Therefore, an omega-3 supplement should be added to a MLG diet in patients with metabolic syndrome.

